# *Pneumocystis* pneumonia in COVID-19 patients: A comprehensive review

**DOI:** 10.1016/j.heliyon.2023.e13618

**Published:** 2023-02-10

**Authors:** Elahe Sasani, Fares Bahrami, Mohammadreza Salehi, Farzad Aala, Ronak Bakhtiari, Alireza Abdollahi, Bahareh Bashardoust, Mahsa Abdorahimi, Sadegh Khodavaisy

**Affiliations:** a Infectious and Tropical Diseases Research Center, Hormozgan Health Institute, Hormozgan University of Medical Sciences, Bandar Abbas, Iran; b Zoonoses Research Center, Research Institute for Health Development, Kurdistan University of Medical Sciences, Sanandaj, Iran; c Department of Parasitology and Mycology, Faculty of Medicine, Kurdistan University of Medical Sciences, Sanandaj, Iran; d Research center for antibiotic stewardship and antimicrobial resistance, Tehran University of Medical Sciences, Tehran, Iran; e Department of Infectious Diseases and Tropical Medicine, Imam Khomeini Hospital Complex, Tehran University of Medical Sciences, Tehran, Iran; f Department of Pathobiology, School of Public Health, Tehran University of Medical Sciences, Tehran, Iran; g Department of Pathology, Imam Khomeini Hospital Complex, Tehran University of Medical Sciences, Tehran, Iran; h Department of Microbiology, Shahr-e-Qods Branch, Islamic Azad University, Tehran, Iran; i Department of Medical Parasitology and Mycology, School of Public Health, Tehran University of Medical Sciences, Tehran, Iran

**Keywords:** Pneumocystosis pneumonia, COVID-19, SARS-CoV-2

## Abstract

The admitted patients of intensive care units with coronavirus disease 2019 (COVID-19) meet the challenges of subsequent infections. Opportunistic fungal infections such as *Pneumocystis* pneumonia (PCP) are among the important factors in the context of COVID-19 patients affecting illness severity and mortality. We reviewed the literature on COVID-19 patients with PCP to identify features of this infection. Although studies confirmed at least the presence of one immunosuppressive condition in half of PCP patients, this disease can also occur in immunocompetent patients who developed the immunosuppressive condition during Covid-19 treatment. The major risk factors associated with COVID-19 patients with PCP can be considered low lymphocyte counts and corticosteroid therapy. Diagnostic and treatment options are complicated by the overlapping clinical and radiologic characteristics of PCP and COVID-19 pneumonia. Therefore, physicians should comprehensively evaluate high-risk patients for PCP prophylaxis.

## 1 Introduction

Cases of coronavirus disease (COVID-19) have spread rapidly worldwide since 2019, causing a public health emergency [1,2]. One of the most common complications in patients with severe COVID-19 pneumonia is acute respiratory distress syndrome (ARDS), requiring intensive care units(ICU) hospitalization, intubation, and mechanical ventilation (MV) [3,4]. To encounter narrowing of the inflammatory airway and consequent cytokines releasing syndrome (CRS), systemic steroids and immunomodulators like tocilizumab, a humanized anti-IL-6 receptor antibody, are diagnosed for these patients [5]. Antiviral immune activation in COVID-19 patients' lung tissue can create an ideal environment for secondary infections and/or coinfections caused by other respiratory viruses such as influenza, bacteria, and fungal (yeasts and filamentous fungi) pathogens [[6], [7], [8], [9]]. COVID-19 associated secondary fungal infections have been shown to significantly impact the severity of the illness and mortality rate [[10], [11], [12]]. Various studies are reported on patients with COVID-19 infection developing opportunistic fungal diseases like candidiasis, pulmonary aspergillosis, and mucormycosis [[13], [14], [15], [16]]. *Pneumocystis* pneumonia (PCP) is an opportunistic infection caused by *Pneumocystis jirovecii*. Its clinical pattern Based on host immune status can change from colonization to cause life-threatening pneumonia [17]. In PCP patients, the challenges due to typically nonspecific clinical, radiological signs and diagnostic difficulties are combined with the possibility for colonization, frequent co-infection with other respiratory pathogens, and low access to sensitive and exact diagnostic tools. Considering poor specificity of clinical PCP definitions in COVID-19 patients, there is a need to establish more robust prevalence estimates, focusing on laboratory-confirmed *P. jirovecii* in respiratory samples from COVID-19 patients [[13], [14], [15], [16]]. To address these gaps, we conducted a comprehensive review to determine the prevalence, diagnostic methods, and treat cases of COVID-19-associated PCP (CAPCP).

## 2 Method

We performed the literature review to better understand the commonalities between related previous investigations using PubMed/MEDLINE, Scopus, and Web of Science databases for published articles from the beginning of 2020 to December 2021. “*Pneumocystis*”, “Pneumocystosis”, “PJP”, or “PCP” were used as mesh keywords, along with “SARS-COV-2″, or “COVID-19". Moreover, the relevant references were manually searched. Molecular-confirmed cases of COVID-19 were included in this review, and articles without the details of CAPCP cases were excluded. From selected studies, the demographic data of the country, age, gender, and the clinical data of underlying disease, CD4 cell count, use of systemic steroids, use of MV, ARDS, ICU admission, anti-COVID-19, anti-PCP treatment, method of PCP diagnosis, and disease outcome were extracted. According to the European Organisation for Research and Treatment of Cancer and the Mycoses Study Group (EORTC/MSGERC) definitions of invasive fungal disease in patients, PCP is categorized as probable, and proven PCP [17,18]. Proven PCP was diagnosed according to radiologic and clinical features plus microscopic observations of *P. jirovecii* on tissue or respiratory samples using conventional or immunofluorescence staining. The diagnosis of probable PCP was based on clinical and radiologic features and relevant host factors, plus the detection of *P. jirovecii* DNA amplified by real-time PCR on respiratory samples and/or 1,3- beta- D-glucan (BDG) in the serum sample.

## 3 Results

### 3.1 Search results and demographic data

**Fig. 1:**
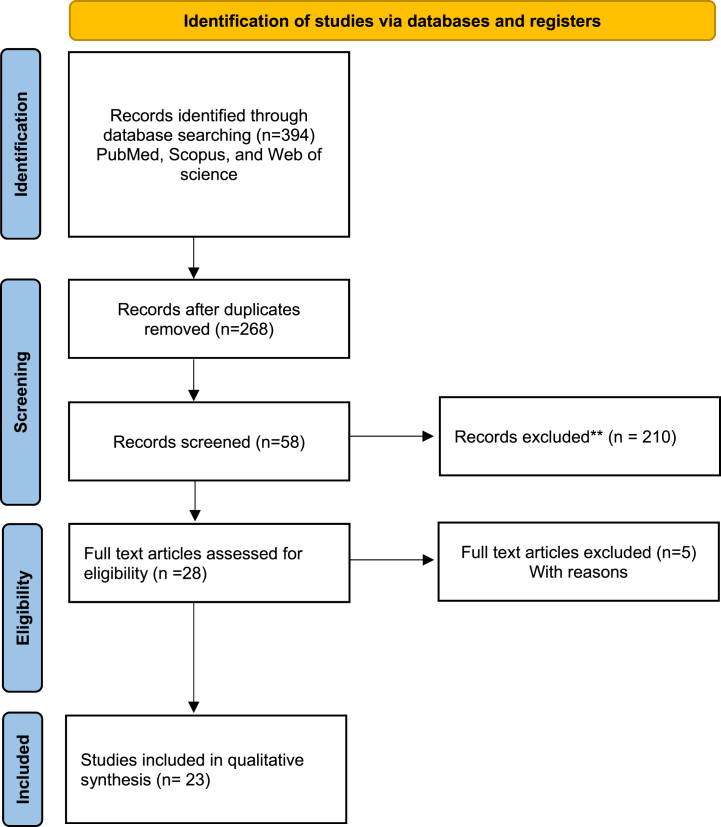
The PRISMA flow diagram presents the search and study selection strategy.

In the initial search, a total of 394 articles were identified by the search in three databases: PubMed (n = 103); Scopus (n = 193); and Web of Science (n = 98). Among them, duplicate articles (n = 126) were removed. After evaluating the titles and abstracts, 210 articles without inclusion criteria were excluded. Finally, 28 studies with the inclusion criteria were reviewed deeply and 23 articles were eligibility [[19], [20], [21], [22], [23], [24], [25], [26], [27], [28], [29], [30], [31], [32], [33], [34], [35], [36], [37], [38], [39], [40], [41]]. Fig. 1 Shows the PRISMA flow diagram of the search and study selection strategy. Table 1 shows a summary of the detailed data of 30 CAPCP cases in the 23 studies including three case series and 20 case reports. The country with the highest number of reported cases was Italy (n = 7/30). Although one case had unknown age, the average age of range was 53.65 ± 19.19 years within a range of 11–83 years. Most CAPCP cases were male [83.3% (25/30)].

**Table 1:** Summary of the demographic and clinical characteristics of COVID-19 patients with-PCP.

No.	Study (country)/case	Age/gender	Underlying disease	CD4 count in HIV (cells/μL)	Proven/Probable in without HIV	Long term Steroid therapy	ICU	MV	ARDS	Anti-COVID-19	Radiological data	LDH (IU/L)	Method of PCP diagnosis/Sample	Other respiratory pathogens	PCP-Treatment, Outcome
1	Bhat P. (USA) [19]	25/M	HIV	32	Probable	Yes	Yes	IMV	–	Remdesivir	GGO, Cystic, pneumothorax	–	PCP antigen testing on BAL	–	TMP/SMX, prednisone Alive
2	Broadhurst A.G.B. (South Africa.) [20]	54/M	HIV, HTN, DM, TB, left ventricular hypertrophy	26	Proven	Yes	Yes	IMV	–	–	GGO	–	Sputum positive PCP DFAT, BDG >500 pg/ml	M.Tuberculosis	TMP/SMX, DEX Death
3	Cai S. (China) [21]	72/F	RA	–	Probable	Yes	–	IMV	–	Tocilizumab, Oseltamivir phosphate, LPV-RTV, Ganciclovir sodium	GGO, Acute consolidation	–	PCP sequencing	A. fumigatus	CAS Alive
4	Coleman H. (UK) [22]	55/M	HIV, asthma	422	Probable	Yes	–	HFNC	–	Emtricitabine, Tenofovir disoproxil, Raltegravir	GGO, Cystic	–	PCP-DNA detection on sputum	–	TMP/SMX, prednisolone Alive
5	Guo W. (the first case) [23]	-/M	HIV	–		Yes	–	NIMV	–	–	GGO	–	CS, low CD4 count, response to TMP/SMZ. method of detection not specific	–	TMP/SMX Then Clindamycin Alive
6	Mang S. (Germany) [24]	52/M	HIV, CMV	12	Probable	Yes	Yes	IMV	–	Darunavir, Ritonavir, Tenofovir/Emtricitabine	GGO, Crazy paving	515	BAL fluid positive for PCP, method of detection not specific	*S. aureus*, *P. aeruginosa*, A.dijkshoorniae	TMP/SMX, prednisone Alive
7	Mouren D. (France) [25] (Case report)	65/M	CLL	–	Proven	NR	–	–	–	–	GGO	–	Positive PCP qPCR from BAL fluid, 2% PCP Cysts by specific silver stains (but not DFAT) on serum, BDG: 80 pg/mL on serum	–	TMP/SMX Alive
8	Rubiano C. (USA) [26] (Case report)	36/M	HIV, HSV-1	<10	Proven	Yes	–	IMV	–	Remdesivir	GGO	789	positive *Pneumocystis* DFA from tracheal aspirate and PCR tests, BDG >500 pg/ml	–	TMP/SMX, prednisone Death
9	Menon A.A. (USA) [27] (Case report)	83/F	Mitral valve prolapse,asthma, ulcerative colitis	291	Probable	NR	–	IMV	–	Azithromycin- Amoxicillin-Clavulanate	GGO, Cystic, Consolidation	348	BDG:305 pg/mL, positive PCP PCR from tracheal aspirate	–	TMP/SMX Alive
10	De Francesco M.A. (Italy) [28] (Case report)	65/M	KT, HTN, DM	–	Probable	Yes	Yes	IMV	Yes	IVIG, Darunavir/ritonavir, HCQ, Tocilizumab, Azithromycin, Piperacillin	GGO	380	Sputum positive for PCP Real-time PCR	A. fumigatus	TMP/SMX,mPRED, DEX Death
11	Farinacci D. (Italy) [29] (Case report)	59/M	HIV, mechanical mitral prosthesis	10	Probable	Yes	Yes	IMV	–	HCQ, Enoxaparin	GGO	645	BDG >500 pg/mL, sputum positive for PCP molecular testing	–	HCQ, Enoxaparin Death
12	Quintana-Ortega C. (Spain) [30] (Case report)	11/F	Anti-MDA5 JDM, RP-ILD	–	Probable	Yes	Yes	IMV	Yes	HCQ, Remdesivir, Tocilizumab	GGO	–	Sputum positive for PCP Real-time PCR	–	TMP/SMX, mPRED, DEX Death
13	Anggraeni A.T. (Indonesia) [31] (Case report)	24/M	HIV	16		Yes	–	NIMV	–	remdesivir	GGO	–	RF, low CD4 count, method of detection not specific	–	TMP/SMX Alive
14	Jeican I·I. (Romania) [32] (Case report)	52/M	chronic smoker and drinker, HTN, chronic alcoholic liver disease, ischemic heart disease	–	Proven	Yes	Yes	IMV	–	–	Consolidation	–	Postmortem (PCP Cysts by Giemsa, GMS, PAS stains on autopsy lung tissue)	–	Death
15	Merchant E.A. (USA) [33]	38/M	CMV, HIV	–	Proven	Yes	–	HFNC	–	remdesivir	GGO	522	BAL positive for PCP by IF	–	TMP/SMX Death
16	Skonieczny P. (Poland) [34] (Case report)	47/M	KT	–	Probable	NA	–	–	–	–	GGO	–	PCP antibody (IgG, IgM) testing on BAL	–	TMP/SMX, Prednisone Alive
17	Blanco J.L (Spain). [35] (Case report)	31/M	HIV	13		Yes	Yes	IMV	–	Dolutegravir, HCQ, Interferon beta,	pleural effusion	1149	RF, low CD4 count, method of detection not specific	–	TMP/SMX, corticosteroids Alive
18	Viceconte G (Italy) [36]^.^ (Case report)	50/M	NO	NR	Proven	Yes	yes	NIMV		dexamethasone, ceftaroline, prednisone	GGO, Consolidation	440	BAL positive for PCP DFAT	–	TMP/SMZ- mPRED Alive
19	Nguyen H· (UK) [37] (Case report)	29/M	XLA with SCIg	NR	Probable	Yes		HFNC		remdesivir, dexamethasone	GGO	–	BAL positive for PCP, method of detection not specific	–	clindamycin and primaquine Alive
20	Peng (Chine) [39] (Case report)	55/M	KT, CVD	292	Probable	Yes	Yes	HFNC		opinavir/ritona vir, Mpred	Consolidation	264	positive Pneumocystis qPCR on sputum	–	TMP/SMZ- Alive
21	Gerber V. (France) [38] (Case series)	80/F	RA	–	Probable	Yes	Yes	IMV	Yes	–	GGO, Consolidation, Nodules	–	PCR on BAL	–	TMP/SMX Death
		70/M	Renal cell carcinoma, HSCT, CKD, PCP six month ago	–	Probable	Yes		IMV	Yes	–	GGO, consolidation	811	positive *Pneumocystis* PCR on tracheal aspirate	–	TMP/SMX Death
		83/M	DM, chronic heart failure, CKD, atrial fibrillation, asthma	–	Proven	NO		NIMV	NO	–	GGO, Crazy paving	437	positive *Pneumocystis* PCR and cytology (PAS) on BAL	–	TMP/SMX Death
		80/M	DM, HTN, transitory, ischemic attack	–	Probable	Yes		IMV	Yes	–	GGO, Pleural effusion	267	positive *Pneumocystis* PCR on tracheal aspirate	–	TMP/SMX, then atovaquone Death
22	Gentile (Italy) [40] (Case series)	68/M	NO	141		Yes	Yes	HFNC	–	prednisone, dexamethasone, enoxaparin	GGO	300	RF, CS, method of detection not specific	–	TMP/SMZ, mPRED Alive
		63/F	HTN, NHL	93	Proven	Yes	Yes	NO	–	remdesivir	GGO		BAL positive for PCP Direct IF	–	TMP/SMZ, prednisone Alive
		55/M	NO	62	Proven	Yes	yes	NIMV	–	dexamethasone, enoxaparin	GGO	338	BAL positive for PCP direct IF	–	TMP/SMZ, mPRED Alive
		75/M	CIHD, persistent atrial fibrillation, DM	1012		Yes	NO	NIMV	–	prednisone, remdesivir, enoxaparin	GGO, Pleural effusion	280	RF, low CD4 count, method of detection not specific	–	TMP/SMZ, mPRED Alive
23	Kronsten (UK) [41] (Case series)	28/M	DM, IgA deficiency, LT		Probable	Yes	Yes	IMV	–	DEX, mPRED, remdesivir	GGO	495	Positive BAL PCR	–	TMP-SMX, switched to primaquine and clindamycin Alive
		51/M	CKD, LT		Probable	Yes	Yes	IMV	–	Prednisolone	GGO		Positive BAL PCR	–	TMP-SMX, switched to primaquine and clindamycin Alive

**Abbreviations:** Anti-MDA5 JDM; Anti-melanoma differentiation-associated gene 5 juvenile dermatomyositis, ARDS; Acute respiratory distress syndrome, A. fumigatus; Aspergillus fumigatus, A. dijkshoorniae; Acinetobacter dijkshoorniae, BAL; Bronchoalveolar lavage, BDG; 1,3-beta-D-glucan, CAS; Caspofungin, CKD; Chronic kidney disease, CLL; Chronic lymphocytic leukemia, CMV; Cytomegalovirus, COVID-19; Coronavirus disease 2019, CS; Clinical symptoms, DEX; Dexamethasone, DFAT; Direct fluorescent antibody test, F; Female, DM; Diabetes mellitus, GMS; Grocott-Gomori's methenamine silver, HCQ; Hydroquinone, HFNC; High-flow nasal cannula, HIV; Human immune deficiency virus, HSCT; Hematopoietic stem cell transplantation, HSV.1; Herpes simplex virus-1, HTN; Hypertension, ICU; Invasive unit care, IMV; Invasive mechanical ventilation, IVIG; Intravenous immunoglobulin, KT; Kidney transplantation, LDH; Lactate dehydrogenase, LPV-RTV; Lopinavir and ritonavir, M; Male, M.Tuberculosis; Mycobacterium Tuberculosis, MV; Mechanical ventilation, NIMV; Non-invasive mechanical ventilation, mPRED; Methylprednisolone, PAS; Periodic acid–Schiff, *P. aeruginosa*; *Pseudomonas aeruginosa*, PCP; Pneumocystis pneumonia, PCR; Polymerase chain reaction, RA; Rheumatoid arthritis, RF; Radiological findings, RP-ILD; Rapidly progressive interstitial lung disease, *S. aureus*; Staph aureus, TB; Tuberculosis, -; Not reported.

### 3.2 Predisposing factors

Ten cases out of 30 (33.3%) had HIV infection. Three cases required extracorporeal membranes oxygenation (ECMO), one of the major complications (16.6%, n = 5) was ARDS, indicating that mentioned patients can be considered severe cases of COVID-19 patients. In addition, corticosteroids were utilized for 83.3% (25/30) of the cases to treat pneumonia or the underlying illness.

### 3.3 Clinical and paraclinical findings

**Fig. 2:**
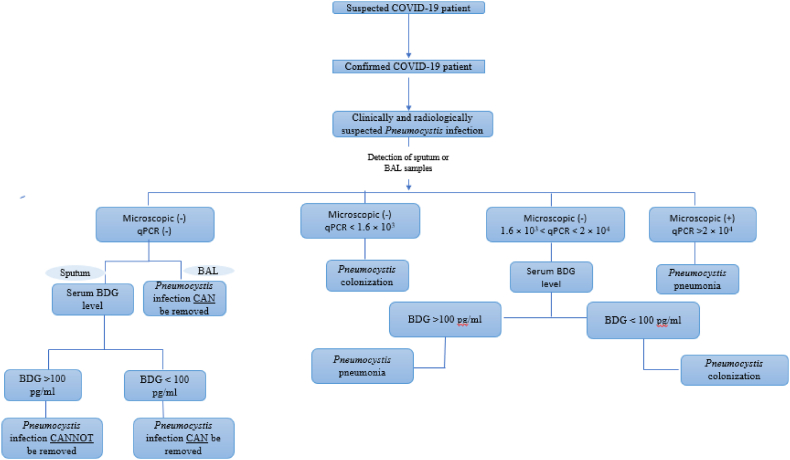
Shows a flow diagram to diagnose *Pneumocystis* infection in COVID-19 patients with clinically suspected PCP. Abbreviations: BAL; Bronchoalveolar lavage, BDG; β-D-glucan.

Ground-glass opacity (GGO) was the commonest radiologic finding 90% (27/30) that identically was reported between PCP and COVID-19 pneumonia. Further, the first and 4th CAPCP cases had cystic lesions, which were based on the distinctive PCP radiological findings. The most common samples used were Bronchoalveolar lavage (BAL) 43.3% (13/30) and sputum 20% (6/30). In most CAPCP cases, molecular approaches 50% (15/30) and microscopic observations 30% (9/30) were used to diagnose *P. jirovecii*. The mean serum BDG level was measured 377 pg/mL in 16.6% of cases (5/30). The mean serum BDG level was measured 377 pg/mL in 16.6% of cases (5/30). Overall, serum lactate dehydrogenase (LDH) was evaluated with a mean of 498.75 IU/L in 53.3% of cases (16/30). The mean value of LDH evaluated (724 IU/L) in cases with HIV (5/30) was almost double in comparison without HIV cases (11/30). The postmortem was diagnosed in one of the cases. Fig. 2 shows a flow diagram to diagnose *Pneumocystis* infection in COVID-19 patients with clinically suspected PCP. According to the EORTC/MSGERC definitions of invasive fungal disease in patients without HIV, 30% of patients (9/30) met the proven PCP criteria.

### 3.4 Treatment and outcome

The remdesivir (n = 6), hydroxychloroquine (n = 4), and tocilizumab (n = 3) were used more than other anti-COVID-19 treatments. The majority of CAPCP cases [90% (27/30)] administrated anti-PCP treatment of the trimethoprim-sulfamethoxazole (TMP-SMX) in addition to steroid therapy of prednisolone, prednisone, methylprednisolone, or dexamethasone. The administration of anti-PCP treatment in three cases was unspecific. Furthermore, the mortality rate among our studied cases was 36.6% (n = 11/30), with the following causes of death: multi-organ failure (12th and 10th cases), especially developed respiratory failure (2nd and 10th instances), and progressive asystolic cardiac arrest (8th case).

## 4 Discussion

### 4.1 Demographic data

In severe diseases, immune disorders can increase the risk of secondary infections with a significant impact on the patients' life quality and survival [12]. According to the previous reports, the risk of secondary fungal infections such as invasive pulmonary aspergillosis (IPA), invasive mucormycosis, or invasive candidiasis in patients with COVID-19 infection has been investigated [10,42,43]. Although among patients with COVID-19 pneumonia, the most common fungal respiratory pathogen is *Aspergillus* spp. [44]. Reports on *P. jirovecii*, the causative agent of PCP, were recently emerging. Among 30 analyzed CAPCP cases in the current study, males (83.3%) were dominant. Similarly, reviews of Ahmadikia et al. and Lai et al. indicated that males were 85.7% and 82.4% COVID-19 patients with mucormycosis and pulmonary aspergillosis, respectively [43,44]. The patients' mean age was <60 years (exactly 53.65 years old) in this review, which validated Ahmadikia et al.‘s review of the COVID-19-associated mucormycosis [43]. Meanwhile, in the previous studies on COVID-19 associated aspergillosis pulmonary, 62.5 years, 63 years, and 66.5 years were reported as the mean ages [10,45,46]. As a result, it is possible to speculate that secondary fungal infections, particularly PCP, in COVID-19 patients are unaffected by age. Gender, on the other hand, can be an effective parameter.

### 4.2 Predisposing factors

HIV disease, organ transplant, diabetes mellitus, and hematologic malignancies can be underlying causes of invasive fungal infection (IFI) [47]. Most cases (90%) evaluated in this study had at least one comorbidity. The most common underlying condition was HIV infection, which accounted for 33.3% (10/30). In HIV patients, PCP is begun gradually and insidiously with few clinical or radiological presentations. Although, in patients with immune disorders without HIV, clinical symptoms with a tendency to the acute and rapid onset of respiratory lead to respiratory failure, and high rates of ICU admissions [15,[48], [49], [50], [51]]. This disparity can be attributable to the severity of pneumonia and the degree of lung inflammation. Furthermore, HIV patients have a higher burden of *P. jirovecii* and fewer neutrophile counts than non-HIV patients [52]. According to the EORTC/MSGERC, solid organ transplantation, glucocorticoid or T-cell suppressive medication use, and a CD4^+^ count of <200 cells/mm3 are risk factors for developing PCP in individuals without HIV [18]. Although the link between PCP and non-immunocompromised ICU patients less attention has been paid, patients with Influenza comprised 7% of reported coinfections [53]. Also, lymphopenia (the decrease of absolute CD4^+^ count and CD4/CD8 ratio) is a similar characteristic in COVID-19 and HIV patients that can be attributed to infection severity [54,55]. Steroid therapy, advised against moderate to severe viral pneumonia, can be a double-edged sword: patients may be saved from viral pneumonia, and can cause secondary fungal and bacterial infections [56]. According to Verweij et al.*'s* systematic review and meta-analysis, patients who take systemic steroids have a greater death risk than those who take a placebo [57]. In addition, systemic steroids were given in 25 instances (out of 30) to treat pneumonia or underlying illness. Immunomodulators have a positive impact on COVID-19 treatment. However, clinicians should keep the risk of taking them for PCP in mind. For example, the medicine tocilizumab, one of the utilized treatments for COVID-19, has been linked to PCP, which was used to treat inflammatory illnesses, including rheumatoid arthritis. Tocilizumab was given to three out of 30 (10%) cases in our study. MV is another predisposing factor in patients with IFIs and severe viral pneumonia, such as COVID-19 [56,58]. For the highest respiratory support, 46.6% (14/30) of COVID-19 cases with PCP required invasive mechanical ventilation (IMV), and 30% (9/30) of them required non-invasive MV (NIMV) and high flow nasal cannula (HFNC), corroborating Chong et al. study [59]. Severe viral pneumonia has a poor prognosis, linked to ICU admission and subsequent fungal infection, resulting in a high fatality rate [58]. In our reviewed cases, sixteen patients were brought to the ICU with a fatality rate of 50% (8/16). In patients with pulmonary aspergillosis related to COVID-19, Arkel et al. and Koehler et al. found that ICU mortality was 67% (4/6) and 60% (3/5), respectively [45,60]. 16.6% (5/30) had ARDS, indicating low PCP risk among COVID 19 patients with ARDS. Our results agree with previous studies [[61], [62], [63]].

### 4.3 Clinical and paraclinical features

Overlapping clinical features between PCP and COVID-19 pneumonia leads to difficulty distinguishing between both pneumonias [64]. This resemblance can be explained by similarities in pathogenic processes of pneumonia produced by *P. jirovecii* and SARS-COV-2, as well as the interaction of both agents with pulmonary surfactant [65]. Further, the common radiologic finding of PCP and COVID-19 pneumonia is GGO, making it difficult to differentiate based on radiological findings [[66], [67], [68], [69]]. One-third of patients with advanced PCP can form cystic lesions [66]. Also, 2/30 (6.6%) of our reviewed cases had these lesions, which helps with other differential diagnoses [19]. COVID-19 patients with PCP can make diagnosis challenging. Although the diagnosis of COVID-19 on nasopharyngeal swabs is rapid and available, the PCP diagnosis is less ordinary [70,71]. BAL fluid was considered the proper sample for diagnosis of PCP because of greater sensitivity [72]. However, due to the danger of SARS-CoV-2 aerosolization, obtaining BAL specimen via bronchoscopy, an invasive and hazardous procedure, cannot be suitable for patients with severe hypoxia [73]. To diagnose definitive PCP, microscopic observations of *P. jirovecii* on respiratory samples by conventional stains (silver stains, toluidine blue) and Immunofluorescent staining with high sensitivity are considered the standard gold test [74,75]. Because laboratories lack either nucleic acid amplification test (NAAT) or immunofluorescent staining, conventional stains can be employed to observe cystic/trophic formations in some specimens, such as histology and cytology [17]. The high charge for the fluorescent microscope is one of the important limitations of IFAs. Significantly, microscopic *P. jirovecii* observations on different respiratory specimens are considered the criterion for proven PCP, while negative microscopical results because of low sensitivity do not exclude infection [17]. NAAT-based methods with more sensitivity than microscopic methods easily do not permit to distinguish between infection and colonization of *P. jirovecii*. Thus, the interpretation of PCR results requires quantifying the fungal load [17]. For PCP detection, qualitative PCR tests, such as conventional and nested are not recommended [17]. Because of the quantitative data and fast speed, real-time PCR is preferred. Positive qPCR results are one of the microbiological criteria for diagnosing PCP, however, negative results do not rule it out. Due to the invasive BAL sampling in severe COVID-19 patients, relevant clinical factors and radiological features together with the serum levels of BDG can be helpful to begin empirical treatment against PCP [76]. Because of the lack of BDG polysaccharides in the COVID-19 virus, the serum BDG level of COVID-19 patients is low (<80 pg/mL) [77,78]. The sensitivity and specificity of serum BDG in the patient with PCP were reported 94.8%, 86.3% in patients with relevant risk factors and clinical signs, respectively [79]. The *P. jirovecii* colonization in COVID-19 patients is prevalent, creating more diagnostic challenges [26]. Due to PCR-based methods or different immunosuppression levels in patients studied, *P. jirovecii* colonization varies in different studies [80,81]. Consequently, some studies have proposed using quantitative polymerase chain reaction (qPCR) and serum BDG levels to differentiate PCP and colonization [82,83]. Notably, a cut-off value of qPCR on BAL samples (>1.6 × 10^3^ DNA copies/μl) and the serum BDG levels with a 100 pg/mL threshold can distinguish PCP and colonization with a sensitivity of almost 100% according to HIV status [82]. Therefore, the combination of PCP qPCR with high sensitivity on BAL and serum BDG levels can prevent the requirement of the immunofluorescent assay (IFA). Even positive results of BDG on serum and PCR on BAL or sputum samples and negative microscopic examinations can lead to increasing the clinical suspicion of PCP in symptomatic patients without HIV [84]. Additionally, three previous studies exhibited *P. jirovecii* colonization in COVID-19 patients [62,63,85]. The improvement of respiratory status without anti–pneumocystosis–specific treatment and the lack of relevant predisposing factors were possible reasons for the differentiation between the colonization and infection of *P. jirovecii* in these studies. The increased LDH level in patients with COVID-19 or PCP as a sensitive biomarker but not specificity can distinguish both infections [86,87]. Although, LDH level of non-survivors is higher than survivors for PCP (mean 447 IU/L vs. 340 IU/L; *P* < 0.05) and COVID-19 (mean 521 IU/L vs. 253 IU/L; *P* < 0.01) [86,88]. Another study showed that a cut-off LDH (>450 IU/L) with a sensitivity of almost 100 could diagnose PCP in patients with relevant clinical signs in the context of non- COVID-19 [86]. In reviewed cases, although serum LDH level of (16/30) was measured, five out of 30 (16.6%) had serum BDG evaluated. Additionally, the use of corticosteroids for severe COVID-19 may further delay the diagnosis of co-occurring PCP due to improvement temporarily of severe PCP. One of the practical interventions is antifungal therapy for patients with PCP. However, the majority of available antifungal drugs are inability to treat pneumocystosis. Thus, the disruption of the folic acid pathway, a good treatment target of *Pneumocystis* organisms, inhibits its synthesis and, as a consequence, the synthesis of protein amino acids and DNA nucleotides [89].

### 4.4 Treatment

The combination of TMP and SMX medicines disrupted the folic acid pathway of *P. jirovecii* and had good results in PCP patients. Twenty-seven CAPCP cases were given this combo medication, and 63% survived. Finally, the clinical and radiological similarities between PCP and COVID-19 pneumonia can cause a delay in diagnosis and therapy. Because of the high prevalence of COVID-19 patients with PCP and advanced HIV illness, HIV testing should be routine in all COVID-19 patients who also have PCP. In COVID-19 patients with HIV/AIDS, paying attention to PCP is critical since early identification and treatment can be beneficial.

### 4.4 Limitations of the study

We have reported the *Pneumocystis* infection among COVID-19 patients in our study. One main limitations of this study is a few case reports or case series with laboratory-confirmed *P. jirovecii*.

Furthermore, the distinguishment between PCP and COVID-19 pneumonia due to overlapping clinical and radiological findings is difficult, especially in non-HIV patients.

## Author contribution statement

All authors listed have significantly contributed to the development and the writing of this article.

## Funding statement

This work was supported by 10.13039/501100004484Tehran University of Medical Sciences [1401-3-252-63348] and 10.13039/501100011917Hormozgan University of Medical Sciences, Bandar Abbas, Iran [Grant No. 4010499] and ethic code [IR.HUMS.REC.1401.359].

### Data availability statement

Data included in article/supp. material/referenced in article.

### Declaration of interest's statement

The authors declare no competing interests.
