# Corrigendum to “Pneumocystis pneumonia in COVID-19 patients: A comprehensive review” [Heliyon 9(2) (February 2023) e13618]

**DOI:** 10.1016/j.heliyon.2023.e16025

**Published:** 2023-05-05

**Authors:** Elahe Sasani, Fares Bahrami, Mohammadreza Salehi, Farzad Aala, Ronak Bakhtiari, Alireza Abdollahi, Aleksandra Barac, Mahsa Abdorahimi, Sadegh Khodavaisy

**Affiliations:** a Infectious and Tropical Diseases Research Center, Hormozgan Health Institute, Hormozgan University of Medical Sciences, Bandar Abbas, Iran; b Zoonoses Research Center, Research Institute for Health Development, Kurdistan University of Medical Sciences, Sanandaj, Iran; c Department of Parasitology and Mycology, Faculty of Medicine, Kurdistan University of Medical Sciences, Sanandaj, Iran; d Research center for antibiotic stewardship and antimicrobial resistance, Tehran University of Medical Sciences, Tehran, Iran; e Department of Infectious Diseases and Tropical Medicine, Imam Khomeini Hospital Complex, Tehran University of Medical Sciences, Tehran, Iran; f Department of Pathobiology, School of Public Health, Tehran University of Medical Sciences, Tehran, Iran; g Department of Pathology, Imam Khomeini Hospital Complex, Tehran University of Medical Sciences, Tehran, Iran; h Department of Microbiology, Shahr-e-Qods Branch, Islamic Azad University, Tehran, Iran; i Department of Medical Parasitology and Mycology, School of Public Health, Tehran University of Medical Sciences, Tehran, Iran; j Clinic for Infectious and Tropical Diseases, Clinical Centre of Serbia, Faculty of Medicine, University of Belgrade, Belgrade, Serbia

In the original published version of this article, an author was replaced with another during production. The original author list has been reinstated (given below).

Elahe Sasani, Fares Bahrami, Mohammadreza Salehi, Farzad Aala, Ronak Bakhtiari, Alireza Abdollahi, Aleksandra Barac, Mahsa Abdorahimi, Sadegh Khodavaisy.

The authors apologize for the errors. Both the HTML and PDF versions of the article have been updated to correct the errors.

## Declaration of competing interest

The authors declare no conflict of interest.

